# Spatiotemporal characteristics and determinants of internal migrant population distribution in China from the perspective of urban agglomerations

**DOI:** 10.1371/journal.pone.0246960

**Published:** 2021-02-12

**Authors:** Chunshan Zhou, Ming Li, Guojun Zhang, Jing Chen, Rongrong Zhang, Yongwang Cao

**Affiliations:** 1 School of Geography and Planning, Sun Yat-sen University, Guangzhou, China; 2 School of Public Administration, Guangdong University of Finance and Economics, Guangzhou, China; 3 School of Geographical Sciences, Fujian Normal University, Fuzhou, China; Institute for Advanced Sustainability Studies, GERMANY

## Abstract

Urban agglomerations are fundamental regional units of development and attract large-scale migrant population. Previous studies have only focused on migrant population distribution in major urban agglomerations. Therefore, this study analysed the spatiotemporal characteristics of migrant population distribution in China during 2000–2010 at city level from the perspective of urban agglomerations. The results indicate that urban agglomerations were accumulation areas of migrant population. Numerous people have migrated into 19 urban agglomerations, which has enlarged regional differences in migrant population distribution. The interprovincial migrant population dominated within urban agglomerations, whereas the intraprovincial migrant population dominated outside urban agglomerations. In the future, intraprovincial migration will become the dominant migration mode. The evolution of migrant population distribution pattern in urban agglomerations agrees with classic theories of unbalanced regional development. The determinants of migration in different regions were compared. Results revealed that economic and government driving forces jointly influenced migration; however, economic forces exceeded government forces. Economic forces were more influential within urban agglomerations, whereas government forces played more important roles outside urban agglomerations. Increased income and job opportunities were the core attractions of urban agglomerations. Moreover, with an increase in the urban agglomeration development level, the influence of economic forces increased, whereas that of government forces decreased. The findings provide a deeper understanding of migrant population distribution in China, which will benefit population management across various regions.

## Introduction

With the acceleration of industrialization and urbanization, urban agglomerations, as advanced spatial organizations, have become the main spatial carrier and the core growth pole of China’s regional economic development since the 21st century [1]. The urban agglomeration is a cluster of large, medium, and small-sized cities and towns with compact space and highly integrated economy. Several metropolitan areas or large cities compose the center region of urban agglomeration, which is connected with highly developed infrastructure networks. The characteristics of an urban agglomeration include a high opening level, high density, polycentricity, and strong flows of essential productive factors [2,3]. Although currently, the concept of “urban agglomeration” is a term with Chinese characteristics, and there is no equivalent concept in other countries. The urban agglomeration is developed from a metropolitan area and will evolve to a megalopolis [2].

Migrant population is an essential productive factor that has a considerable effect on economic development and urbanization [1,4,5], and urban agglomerations are spatial carriers of various essential productive factors [2]. Therefore, internal migrant population gather in urban agglomerations [6,7]. The total interprovincial migration in China was 11.07 and 85.88 million in 1990 and 2010, respectively. A total of 4.49 and 61.2 million people migrated in 1990 and 2010, respectively, to three major urban agglomerations (Beijing–Tianjin–Hebei, Yangtze River Delta and Pearl River Delta). The aforementioned numbers accounted for 40.6% and 71.2% of the total interprovincial migrant population in 1990 and 2010, respectively. Because most of the migrant population engages in non-agricultural economic activities and lives in cities or towns, they are counted as urban permanent residents. A total of 74.5% and 83.4% of the interprovincial migrant population moved into cities or towns in 2000 and 2010, respectively. The large-scale migrant population in urban agglomerations brought about a rapid growth in urban permanent residents, which has promoted the urbanisation level of these agglomerations. In 2012, the overall urbanisation ratio in China reached 52.6% and the urbanisation ratio of the aforementioned three major urban agglomerations exceeded 65% [8]. Consequently, studies regarding the spatiotemporal patterns and dynamics of migrant population from the perspective of urban agglomerations are crucial for the establishment of a new urbanisation policy and for regional economic development.

The polarization of migrant population distribution was found to get strengthened during 1990–2010. Main migration destinations are southern and eastern coastal regions, such as Guangdong, Jiangsu, Shanghai and Beijing. Southwest China and Midstream of Yangtze Plain, such as Sichuan, Henan, Anhui and Hunan, were the main migration source regions [6,9]. Migration pattern studies conducted from the perspective of urban agglomerations have focused only on several developed urban agglomerations [8,10–13]. Based on the China Migrants Dynamic Survey in 2015, 66.22% of migrants in Beijing–Tianjin–Hebei urban agglomeration were interprovincial and 77.95% of migrants stayed in three major cities (Beijing, Tianjin and Shijiazhuang) [13]. The spatiotemporal characteristics of migrant population distribution must be comprehensively analysed for all cities within and outside urban agglomerations to conduct appropriate population management. Moreover, factors influencing migrant population distribution within and outside Chinese urban agglomerations have not been compared. Therefore, based on the 5th and 6th population censuses, this study used the Theil index and regression models to analyse the spatiotemporal characteristics and determinants of China’s migrant population distribution from the perspective of urban agglomerations. The findings of this study could provide useful references and policy implications for the development of a new Chinese urbanisation policy and the adjustment of the population regulation policy.

### Economic and government driving forces of migration

The selection of migration destinations by migrants result in the spatial pattern of migrant population distribution. Thus, analysing the determinants of migrants’ destination decisions may indicate the migrant population distribution pattern. According to push and pull migration theory, the determinants of migration can be classified as push and pull forces. Push forces are negative factors that promote migrants to leave their previous residence, and pull forces are positive factors that attract migrants to new regions [6,10]. However, previous studies based on the gravity model have found that pull forces, rather than push forces, dominate China’s internal migration [14–16]. Therefore, this study focused on pull factors.

**Fig 1:**
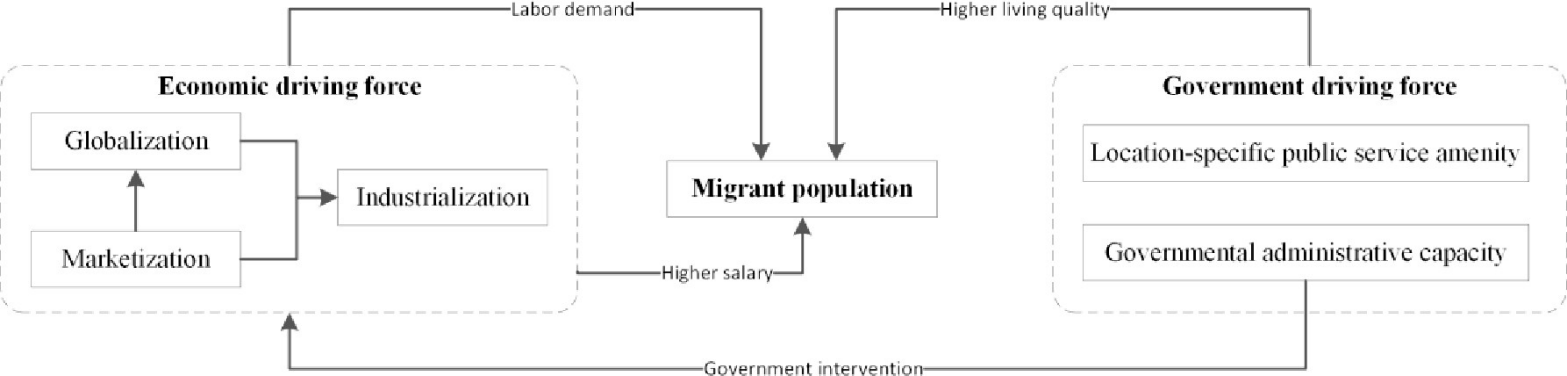
Conceptual framework of economic and government driving forces of migration in China.

As per neoclassical migration theory, many earlier Western studies have considered migration as an economic behaviour, namely a human capital investment based on rational cost–benefit calculation, and economic opportunity as the sole driver of migration. In particular, migrants move in search of increased income and employment opportunities [6,14]. Economic growth brought about job opportunities and increased income, and redistributed the Chinese population under the processes of marketisation, globalisation, and industrialisation (Fig 1) [17–19]. Marketisation laid the foundation for the economic development of China. The nonstate-owned economy transformed the market from egalitarianism to comparative advantage, which caused the massive migration of labour forces [20]. Marketisation also provided a foundation for globalisation because it attracted massive foreign investments. Foreign capital was initially invested in the eastern coastal areas of China. With the deepening of reforms, investments spread to inland areas [21]. The influx of foreign capital promoted the manufacturing industry and created substantial jobs, which attracted a substantial labour force. Foreign investments transformed China into a global factory and enabled financial and technical support to China’s industrialisation [18]. Industrialisation was one of the core drivers of urbanisation in China because it created employment opportunities and offered increased wages [22,23], which attracted a massive surplus of labour and reshaped the population distribution pattern.

Apart from the aforementioned neoclassical theory which regards economic opportunities as the sole driver if migration, migrants were also found to pay attention to urban living qualities [24]. The quality of location-specific public service amenities, such as medical facilities, schools and museums, determine urban living qualities. Restrictions on household registration (Hukou) in China have been gradually lifted [6], and migrants without local Hukou are partially included in these local social welfare system. The location-specific public service amenities in China are mainly managed by the government [6,25]. Therefore, location-specific amenities can be considered as a government force to attract and accommodate migrants. Moreover, the government can regulate economic development to a certain extent through the administrative ability, thus affecting population migration. After the economic reform, China transformed into socialist market economy in which economy mechanisms operated with government intervention [4,17]. The government played a vital role in distributing socio-economic resources, such as construction land quotas and public financial investments. The administrative hierarchy level refers to the governmental administrative capacity. Cities with a high administrative hierarchy level have privileged access to the acquisition of land quotas, key industrial projects, and major infrastructure projects, which can create numerous job opportunities, improve the quality of public service [26], and increase the level of migrant attraction.

Internal migration is common worldwide [27], such as in the United States [28], Russia [29], and Latin America [30]. The migration intensity in Western countries is higher than that in China [27]. In 1981, internal migration rates in North America has reached about 17%-19% [29], and in 2018, internal migration rates in China is 17.27%. Although migration in Western countries is also influenced by socioeconomic factors, such as income, housing price and the quality of public service [28,29], government intervention in population migration is less. Unlike Western market-orientated capitalist economies, China adapts market and central planning mechanisms simultaneously. Remnants of the socialist period, such as the Hukou system and government-oriented infrastructure investments, continue to shape the patterns of economic and social development and population migration [14]. For example, there are many high-level private hospitals and private schools in Western countries, while most of China’s high-level hospitals and schools are public. Therefore, migration in Western countries is considered as a market behaviour and is mainly affected by economic factors. However, as the aforementioned discussion, economic and government driving forces jointly determine internal migration in China (Fig 1), which distinguishes China’s migration from that of Western countries [4,16,20,31]. As crucial supports of regional economic development, urban agglomerations are main accumulation areas for migrants. However, few studies have compared differences in the driving mechanisms of economic and government factors on migrant population growth within and outside urban agglomerations as well as in different urban agglomerations.

## Materials and methods

### Study area and data sources

The prefecture-level city is usually the basic unit of implementation of many policies in China. Therefore, this paper analysed migrant population distribution at the prefecture-level city scale. The accurate national city-level demographic data can only be obtained from national population censuses every ten years. The 1% National Population Sample Survey in 2015 only issues estimated demographic data at provincial level. Therefore, city-level demographic data were obtained from the 5th and 6th population censuses in 2000 and 2010. In this study, people who had resided in one city for more than 6 months but had their household registration (Hukou) in another city at the time of the survey were identified as the migrant population. People whose household registration (Hukou) and residence are in the same prefecture-level city, but not in the same district, county or county-level city are excluded from migrant population. Because in China, the prefecture-level city is usually the basic unit of implementation of many policies, and they are included the same public services system and considered as locals. The migrant population was divided into intraprovincial and interprovincial migrants, which refer to migrants whose current residence and household registration were in the same province and different provinces, respectively.

**Fig 2:**
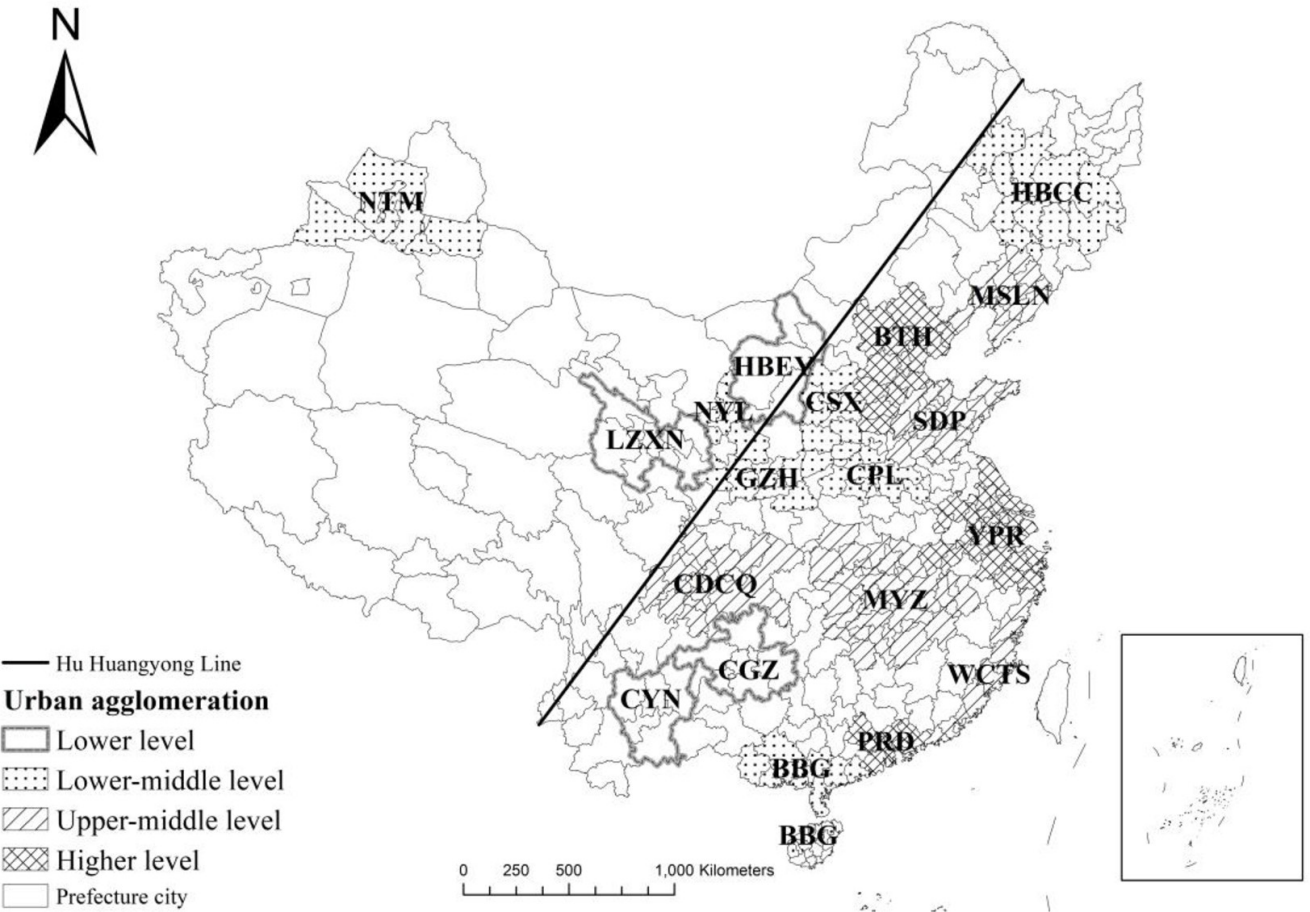
Study area and urban agglomerations (the administrative boundaries of prefecture-level city are republished from [32] under a CC BY license, with permission from the resource and environment data centre, original copyright 2015).

The Hu Huanyong Line splits China into two approximately equal parts. But nearly 94% of the Chinese population lives on the eastern side of the Hu Huanyong Line [7]. The vector data of administrative boundaries of prefecture-level city was provided by the Resource and Environment Data Centre (http://www.resdc.cn/). According to the administrative divisions at the prefecture-city level in 2010, 362 cities were included in this study. Hong Kong, Macao, and Taiwan were excluded from this study due to the lack of data. The 5th population censuse in 2000 was adjust to the administrative division in 2010. A total of 19 urban agglomerations were selected according to the 13th Five-Year Plan (2016–2020) [1]. The boundaries of these urban agglomerations were defined according to their master plans, and mapped in Fig 2 based on the administrative boundaries in 2010. Moreover, socioeconomic data were extracted from the China City Statistical Yearbook 2011 and China Statistical Yearbook for Regional Economy 2011.

### Development level of urban agglomerations

The development levels of 19 urban agglomerations were identified by calculating their development degree indices [1,33]. The development degree indices of urban agglomerations were composed of 14 sub-indexes, covering overall level of economic development, city size, infrastructure conditions, urbanization level, industrial superiority, etc. The weight coefficients of the 14 indexes were consistent with Fang et al. [33]. Socioeconomic data were extracted from the China City Statistical Yearbook 2011 and China Statistical Yearbook for Regional Economy 2011.

The development levels of urban agglomerations were grouped into three main grades: high level, middle level, and low level. The middle level was further divided into lower- and upper-middle levels (Table 1). Urban agglomerations with high and upper-middle levels of development are mainly located in coastal regions and along the Yangtze River. Moreover, high-development-level urban agglomerations are surrounded by urban agglomerations with middle level of development. Urban agglomerations with low level of development are located in the inland midwest region and are far away from high-development-level urban agglomerations (Fig 2).

**Table 1:** Development level of urban agglomerations in China in 2010.

Development level		Total amount	urban agglomeration
Low level		4	Central Yunnan (CYN), Lanzhou-Xining (LZXN), Hu-Bao-E-Yu (HBEY), Central Guizhou (CGZ)
Middle level	Lower-middle level	7	Northern Tianshan Mountains (NTM), Guanzhong (GZH), Central Plains (CPL), Central Shanxi (CSX), Harbin-Changchun (HBCC), Beibu Gulf (BBG), Ningxia Yellow River (NYL),
	Upper-middle level	5	Middle Reaches of Yangtze River (MYZ), Shandong Peninsula (SDP), Chengdu-Chongqing (CDCQ), Western Coast of Taiwan Straits (WCTS), Mid-southern Liaoning (MSLN),
High level		3	Yangtze River Delta (YRD), Pearl River Delta (PRD), Beijing-Tianjin-Hebei (BTH)

### Theil index

The Theil index can measure total inequality as well as within-group and between-group inequality. This index was first used to examine the income gap among different countries [34,35]. In this study, the Theil index measured the inequality of migrant population distribution for reflecting the influence of urban agglomerations on the migration pattern. The overall Theil index and the Theil indices of urban agglomerations at different development levels were calculated. Cities were then divided into two groups, namely cities within and outside urban agglomerations, and we decomposed the overall Theil index into within-group components and between-group components.

$$T=\sum_{i=1}^{n}\frac{P_{i}}{P}\ln(n\frac{P_{i}}{P})=TBG+TWG$$ (1)

$$TBG=\frac{P_{w}}{p}\ln(\frac{n}{n_{w}})+\frac{P_{o}}{p}\ln(\frac{n}{n_{o}})$$ (2)

$$TWG=\sum_{i=1}^{n_{w}}\frac{P_{i}}{p}\ln(n_{w}\frac{p_{i}/p}{p_{w}/p})+\sum_{i=1}^{n_{o}}\frac{P_{i}}{p}\ln(n_{o}\frac{p_{i}/p}{p_{o}/p})$$ (3)

where *T* is the overall Theil index, *n* is the total number of prefecture-level cities, *P*_*i*_ is the number of migrant population of *i* city, *P* is the total migrant population, *TBG* is the between-group Theil index, *TWG* is the within-group Theil index, *P*_*w*_ is the number of migrant population within urban agglomerations, *P*_*o*_ is the number of migrant population outside urban agglomerations, *n*_*w*_ is the number of prefecture-level cities within urban agglomeration, *n*_*o*_ is the number of prefecture-level cities outside urban agglomeration.

### Empirical models and variable specification

Economic and government forces shaped the observed population redistribution patterns. Explanatory variables (Table 2) were selected according to aforementioned conceptual framework (Fig 2) and following previous studies. With regard to economic factors, marketisation paved the road for globalisation [19]. These two processes brought capital and technology for industrialisation, which created a massive number of jobs and was the core driver of economic growth in China [17,18]. Hence, the numbers of employees in private enterprises and self-employed individuals (PIV) were used to determine the marketisation force. The total amount of foreign capital actually utilised (FC) represents the globalisation force [18]. The average number of employees in secondary and tertiary industries (EMP) was used to measure the industrialisation force [18]. The average wage of employees (WAGE) was considered the engine of migration and denotes the level of economic development [9,36].

**Table 2:** Description of considered variables.

	Variables	Description
Dependent variable	MIGPOP	Natural logarithm of migrant population
Economic determinants	EMP	Natural logarithm of average number of employees in secondary and tertiary industry
	WAGE	Natural logarithm of average wage of employees
	FC	Natural logarithm of total amount of foreign capital actually utilized (2001–2010)
	PIV	Natural logarithm of employees in private enterprises and self-employed individuals
Administrative determinants	EDU	Natural logarithm of expenditure for education
	SS	Natural logarithm of expenditure for social safety and employment effort
	HLTH	Natural logarithm of expenditure for medical and health care
	GBX	Natural logarithm of local government other general budget expenditure
	LVL	Administrative hierarchy level; prefecture-level cities = 1; provincial capitals = 2; Cities specifically designated in the state plan = 3; Sub-provincial cities = 4; Municipalities directly under the central government = 5

Although the Hukou system restricts population settlement, population migration is not restricted [6]. Local governments can improve urban living quality and vary the allocation of economic resources to attract migrant population [6,25,26]. Therefore. expenditure for education (EDU), expenditure for social safety and employment effort (SS), expenditure for medical and health care (HLTH), and local government other general budget expenditure (GBX) were selected to measure different types of public services separately [37]. The administrative hierarchy level (LVL) of cities was used to capture the governmental administrative capacity [6,26]. Accordingly, the empirical model was formulated as follows:

$$MIGPOP_{i}=\beta_{0}+\beta_{1}EMP_{i}+\beta_{2}WAGE_{i}+\beta_{3}FC_{i}+\beta_{4}PIV_{i}+\beta_{5}EDU_{i}+\beta_{6}SS_{i}+\beta_{7}HLTH_{i}+\beta_{8}GBX_{i}+\beta_{9}LVL_{i}+\varepsilon_{i}$$ (4)

where *MIGPOP*_*i*_ is the total migrant population in city *i* in 2010 and *β* and *ε* are regression coefficient and error term, respectively. The definitions of EMP, WAGE, PIV, FC, GBX, and LVL are listed in Table 2.

## Results and discussion

**Fig 3:**
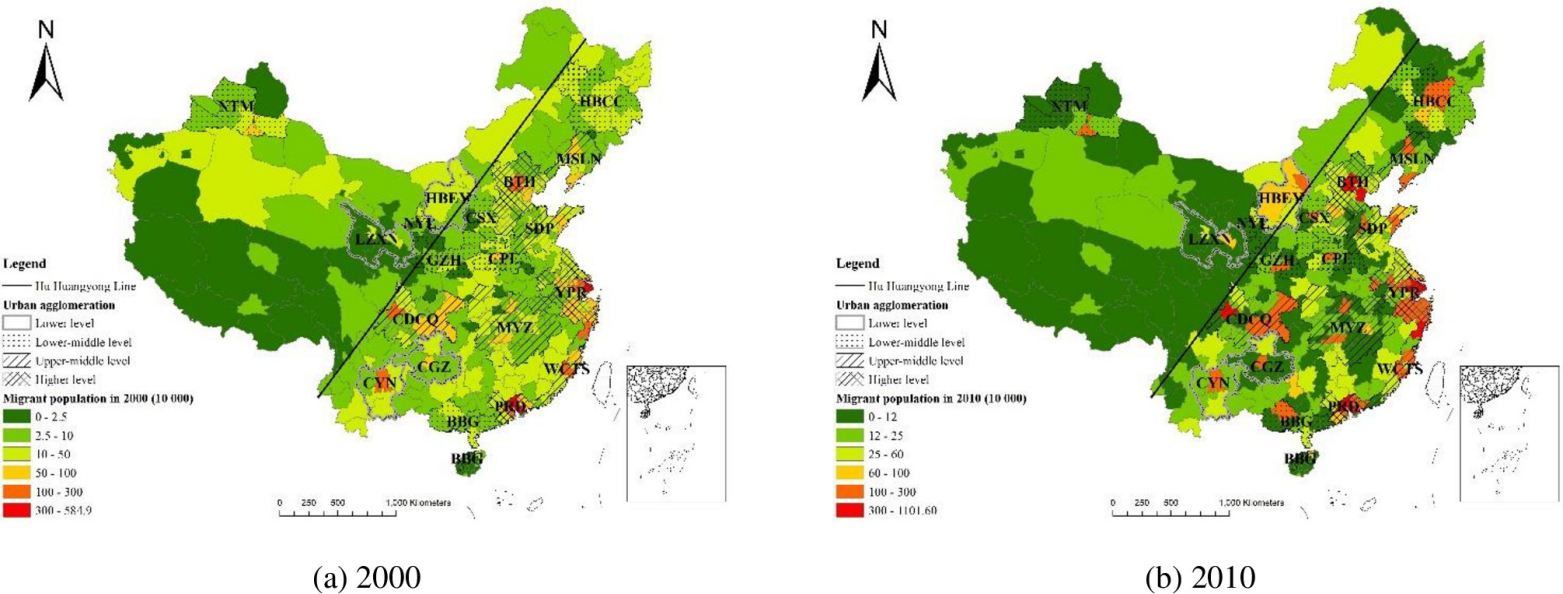
Spatial distribution of the migrant population in China (the administrative boundaries of prefecture-level city are republished from [32] under a CC BY license, with permission from the resource and environment data centre, original copyright 2015).

The number of migrants of each city, obtained from 5th and 6th population censuses, was linked to the vector data of administrative boundaries in 2010 and mapped in Fig 3. The area of each city was calculated based on the vector data of administrative boundaries, and statistics characteristics of the migrant population distribution in China in 2000 and 2010, such as migrant population density and annual growth rate, were listed in Table 3.

**Table 3:** Statistics of the migrant population in China in 2000 and 2010.

	Migrant population (10 000)		Migrant population density (people per 100km^2^)		Annual growth rate of migrant population (%)	Proportion of interprovincial migrant population in total migrant population in area (%)		Proportion of migrant population in total migrant population (%)	
	2000	2010	2000	2010	2000–2010	2000	2010	2000	2010
Nation	7811.69	16955.24	861	1865	8.04	53.87	50.34	100	100
Outside urban agglomeration	971.16	1740.52	160	286	5.97	36.76	32.72	12.46	10.27
Within urban agglomeration	6840.53	15214.71	2290	5086	8.31	56.30	52.35	87.54	89.73
Low level	416.38	905.45	722	1563	8.03	36.94	28.97	5.51	5.51
Lower-middle level	803.96	1830.78	809	1824	8.48	39.32	27.87	10.53	10.96
Upper-middle level	1762.39	4151.44	1906	4486	8.94	41.53	36.23	22.47	24.41
High level	3857.79	8327.04	8494	18334	8.00	68.90	68.54	49.03	48.84

### Spatiotemporal characteristics of migrant population distribution in China from the perspective of urban agglomerations

**Urban agglomerations are accumulation areas of migrant population.** Urban agglomerations are the accumulation areas of migrant population. The total area of the 19 considered urban agglomerations is 3.1 × 10^6^ km^2^, which accounts for 32.75% of the study area. The migrant population in these agglomerations accounted for 87.54% of the total Chinese migrant population in 2000 and 89.73% of the total Chinese migrant population in 2010 (Table 3). Cities with a large amount of the migrant population were mainly distributed to the east of the Hu Huanyong Line, especially in the eastern coastal region, which is in agreement with results obtained in other studies [6,9]. Some core cities of inland urban agglomerations also had large-scale migrant population, such as Chongqing, Chengdu, Nanning (Fig 3). Some cities in inland urban agglomerations, such as Chongqing, Chengdu, and Nanning, were also home to a large migrant population (Fig 3). In 2000, the average density of the migrant population in the 19 urban agglomerations was 2290people/100 km^2^, which was significantly higher than the national average migrant population density (861 people/100 km^2^) and the migrant population density outside urban agglomerations (160 people/100 km^2^). In 2010, the migrant population density in urban agglomerations increased to 5086 people/100 km^2^, the national average migrant population density was 1865 people/100 km^2^, and the migrant population density outside the urban agglomerations was 286 people/100 km^2^. With the development of Information and Communications Technology (ICT), big data, such as mobile phone signalling data and mobile app Location-based services (LBS) data, are used to detect population mobility [38]. Because most migrants return home for family reunion during the Chinese Spring Festival, and population mobility big data during this period can reflect the spatial distribution of migrant population. The YRD, PRD and BTH were also found to be the most important sink areas for migrant population based on dataset during 2016 Chinese Spring Festival [39], which matches our results.

#### The attraction of urban agglomerations enlarges migration differences within and outside of urban agglomerations

The migrant population has continued to flow into urban agglomerations, and the gap between the migrant population within and outside urban agglomerations has been widening. According to the statistical data in Table 3, during 2000–2010, the annual growth rate of migrant population in urban agglomerations was 8.31%, which was higher than the national growth rate (8.04%). During the aforementioned period, the annual growth rate of the migrant population outside urban agglomerations was only 5.97%. Although the annual growth rate of migrants in northeast China was 7.11% during 2000–2010, which was lower than the national average growth rate. However, the annual growth rate of migrant population in urban agglomerations in northeast China was 8.25%, which was still higher than the national rate. The proportion of migrant population in urban agglomerations increased from 87.67% in 2000 to 89.7% in 2010 (Table 3). The aforementioned results highlight the attraction of urban agglomerations to the migrant population.

The overall Theil index of China’s migrant population distribution at city level increased from 1.091 in 2000 to 1.133 in 2010, which indicates that the polarised spatial pattern of migration strengthened during 2000–2010. This polarised pattern is consistent with the previous finding that the migrant population in the top 100 largest Chinese cities was still increasing [6,9]. Cities were divided into two groups: cities within and outside urban agglomerations. The intergroup Theil index increased during 2000–2010. Moreover, the contribution rate of the intergroup Theil index for overall Theil index increased from 14.024% in 2000 to 16.681% in 2010 (Table 4), which indicates that regional differences in the number of migrants within and outside urban agglomerations has widened. Because of rapid economic development, cities in urban agglomerations have more job opportunities, higher wages, and more comprehensive infrastructure systems than cities outside urban agglomerations. These attractions have resulted in a substantial migration of people into urban agglomerations, which has enlarged the imbalance in the distribution of the migrant population in China.

**Table 4:** Theil index of migrant population scale and its decomposition.

	2000		2010	
	Theil index	Contribution rate (%)	Theil index	Contribution rate (%)
National	1.091	100.00	1.133	100.00
Intra-group	0.938	85.976	0.944	83.319
Inter-group	0.153	14.024	0.189	16.681
Low level	0.685	-	0.587	-
Lower-middle level	0.532	-	0.726	-
Upper-middle level	0.626	-	0.795	-
High level	0.810	-	0.718	-

#### Interprovincial migrant population is dominant within urban agglomerations, whereas intraprovincial migrant population is dominant outside urban agglomerations

The migrant population can be divided into two types: intraprovincial and interprovincial. In 2000 and 2010, the proportions of the interprovincial migrant population in China were 53.87% and 50.34%, respectively. This matches the previous finding that the intensity of intraprovincial migration was lower than that of interprovincial migration [9]. In 2000 and 2010, the proportions of the interprovincial migrant population within urban agglomerations were 56.30% and 52.35%, respectively. The proportions of the interprovincial migrant population outside urban agglomerations were only 36.76% and 32.72% in 2000 and 2010, respectively (Table 3). The migrant population within urban agglomerations was mainly interprovincial, and the attraction of cities in urban agglomerations was considerably higher than that of cities outside urban agglomerations.

The higher the development level of urban agglomerations, the stronger is their attractiveness to the migrant population and the higher is their proportion of the interprovincial migrant population. In 2000, the proportions of the interprovincial migrant population in urban agglomerations with high, middle-upper, middle-lower, and low levels of development were 68.90%, 41.53%, 39.32%, and 36.94%, respectively (Table 3). By 2010, although the proportions of the interprovincial migrant population in urban agglomerations decreased for all development levels, the pattern of a high level of development resulting in a high proportion of the interprovincial migrant population still remained. Previous studies also found similar patterns. Liu et al. [6] found that in the eastern coastal region of China, interprovincial migration mainly occurs in high-development-level urban agglomerations. And Chen et al. [13] found that proportions of migrants from outside BTH of Beijing and Tianjin in 2015 (75.33% and 81.31%) were higher than that of BTH (66.22%) and Hebei province (42.57%).

Although interprovincial migration is still the main mode of migration in urban agglomerations, the growth rate of intraprovincial migration is faster than that of interprovincial migration and the proportion of intraprovincial migration gradually increases. A possible explanation for this result is that the interprovincial migrant population encounters more obstacles in the urbanisation process than the intraprovincial migrant population does. Previous studies have found that the intraprovincial migrant population lives longer in host cities and has stronger settlement intentions than the interprovincial migrant population [6,40]. Thus, intraprovincial migration will eventually become the dominant mode of urbanisation in China.

### Effect of urban agglomerations on migrant population distribution

**Fig 4:**
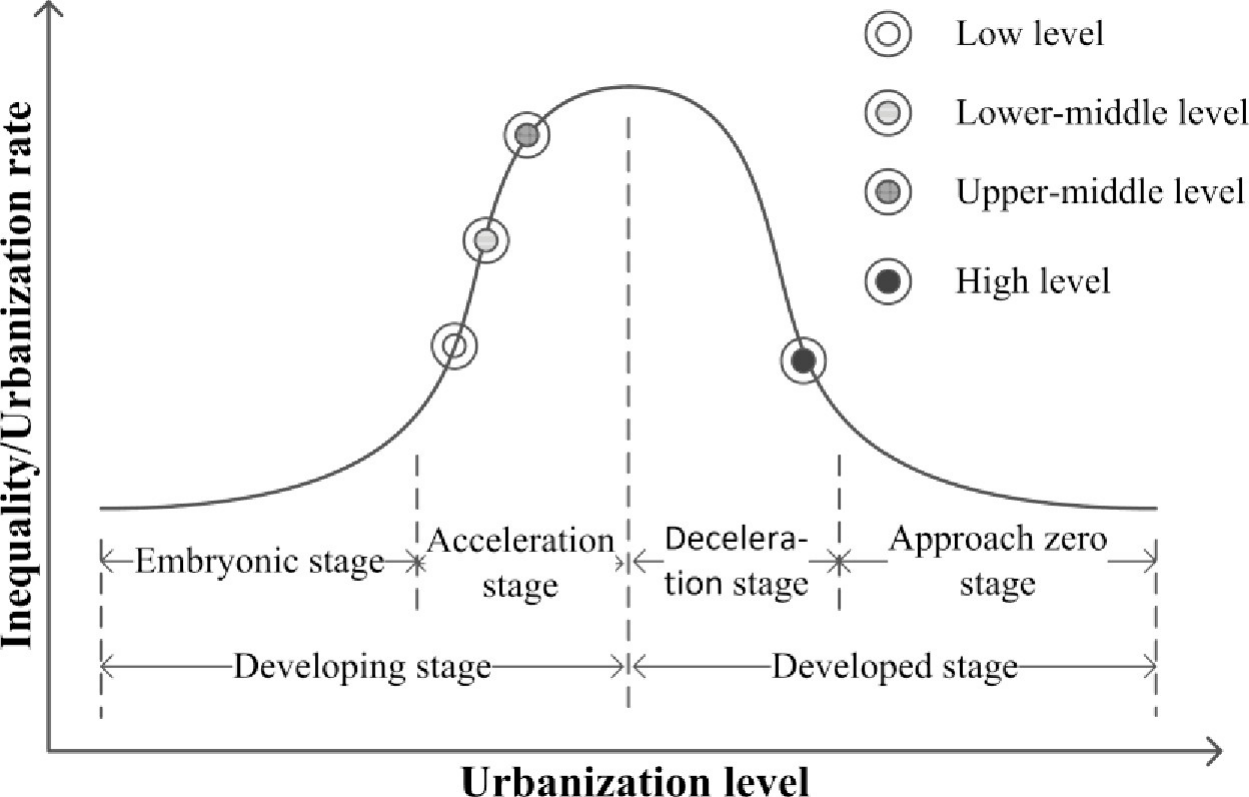
The curve of the urbanisation rate, regional inequality, and urbanisation stage.

According to the aforementioned statistical analyses and data in Tables 3 and 4 and Fig 3, it is found that the spatiotemporal characteristics migrant population distribution in urban agglomerations at different development level agree with classical unbalanced regional development theories, such as Friedmann’s core–periphery theory, Hirschman’s unbalanced growth theory, and Williamson’s inverted-U theory. The regional inequality and urbanisation rate vary in an inverted-U-shape with economic development (Fig 4). In the early development stage, the agglomeration of productive factors promotes the rapid growth of regional centres, which results in the formation of a mono-core structure. In this stage, the urbanisation rate increases and the regional inequality gradually widens. In the developed stage, the diffusion effect promotes the development of surrounding areas, which results in the formation of a balanced and complex multicore structure. In this stage, the urbanisation rate decreases and the regional inequality gradually narrows [11,41]. Migrant population is an index of urbanisation in China [9]. Urban agglomerations at different development levels have different urbanisation levels, and different migrant population distribution pattern. A conceptual model of China’s migrant population distribution was developed from the perspective of urban agglomerations.

**Fig 5:**
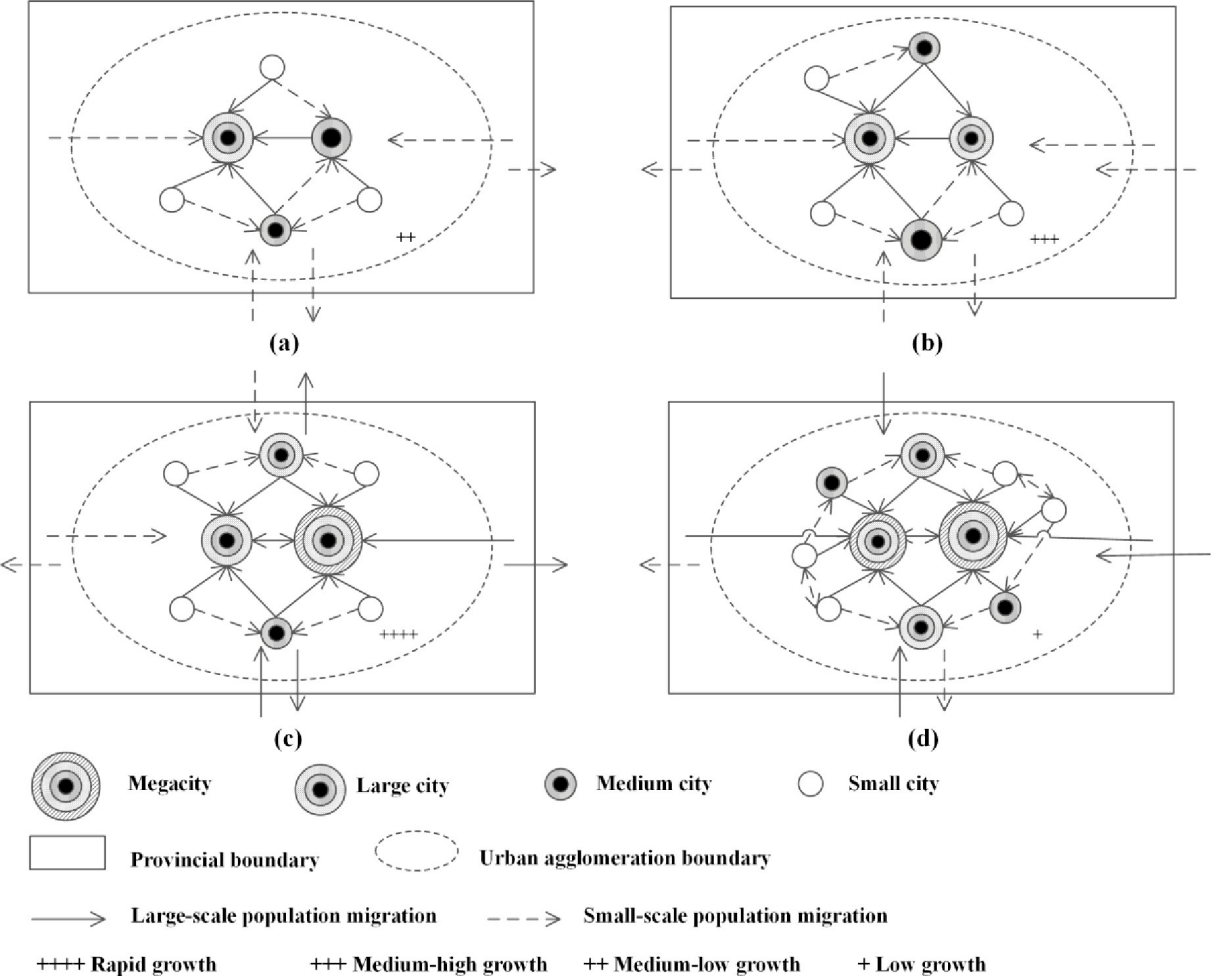
Conceptual model of migrant population distribution in urban agglomerations.

Urban agglomerations with low level of development level just enter the accelerated stage of development when the urbanisation level and rate are relatively low [41]. In low-development-level urban agglomerations, the population and population density of migrants were the lowest. The migrant population growth rate, proportion of the interprovincial migrant population, and Theil index of migrant population distribution were relatively low (Tables 3 and 4 as well as Fig 4). Because of the relatively low regional economic development level, various production factors gathered to individual core city. Population migrated from edge cities and cities outside urban agglomerations to core cities. The population growth rate of core cities was high, and differences in the migrant population of core and edge cities in urban agglomerations began to widen. Urban agglomerations with low level of development are located far away from those with high level of development. Therefore, the population in edge cities within urban agglomerations and cities outside urban agglomerations preferred to migrate to the core city within the same province rather than to a far-away urban agglomeration with high development level. Consequently, a predominance of the intraprovincial migrant population was observed (Fig 5A).

Urban agglomerations with lower-middle level of development were in the mid-acceleration stage of urbanisation, and their urbanisation rates began to accelerate [41]. In urban agglomerations with a lower-middle level of development, the population, population density, and proportion of interprovincial migrants were relatively low. However, the growth rate of migrant population and the Theil index of migrant population distribution were relatively high (Tables 3 and 4 as well as Fig 4). In the mid-acceleration stage of urbanisation, industrialisation began to dominate economic growth. With increased wages available in cities, rural surplus labourers migrated to cities and towns and populations migrated from edge cities to core cities. The size of cities and towns as well as their population increased. With the rapid flow of various production factors to core cities, gaps between core cities and other cities within urban agglomerations widened. Moreover, a sub core city began to emerge in urban agglomerations but was not completely formed. Because urban agglomerations with lower-middle level of development were located close to high-development-level urban agglomerations, their attraction to surrounding cities outside urban agglomerations was relatively weak, and some people chose to migrate to urban agglomerations with a high level of development. Therefore, the migrant population was still dominated by intraprovincial migrants. The regional inequality of migrant population distribution thus widened (Fig 5B).

Urban agglomerations with upper-middle level of development were in the final accelerated stage of urbanisation, and their urbanisation rate reached the peak value. Industrial development entered a mature stage, and the economy was growing at a high speed [41]. In urban agglomerations with upper-middle level of development, the population, population density, and proportion of interprovincial migrants were relatively high. The growth rate of migrant population and the Theil index of migrant population distribution were the highest for these urban agglomerations (Tables 3 and 4 as well as Fig 4). A substantial number of rural surplus workers migrated to cities. Labour influxes to all cities within the aforementioned urban agglomerations increased. Moreover, the migrant population of most cities within urban agglomerations with upper-middle level of development continued to increase. When the growth rate of migrants in core cities increased, a sub core city was formed. Links among cities within the aforementioned urban agglomerations were strengthened, and the regional inequality of migrant population distribution widened. However, because urban agglomerations with upper-middle level of development were located close to high-development-level urban agglomerations, the outflows of population were substantial (Fig 5C).

High-development-level urban agglomerations were in the deceleration stage of urbanisation, and their speed of urbanisation was decreasing [41]. These urban agglomerations had the highest migrant population and migrant population density, and the interprovincial migrant population was dominant. However, the growth rate of the migrant population and the Theil index of migrant population distribution were the lowest for the aforementioned urban agglomerations (Tables 3 and 4 as well as Fig 4). Their attraction to the migrant population had reached saturation. In the deceleration stage of urbanisation, the industrial structure was gradually being upgraded and the service industry gradually became a pillar industry. The education level and professional skills of the migrant population improved. The economy was still growing; however, it entered a transitional period. Two or more mega-cities emerged, and a double-centre and secondary polycentric structure was formed (e.g., Hangzhou and Nanjing in YRD, Foshan and Dongguan in PRD). The regional inequality of migrant population distribution was narrowing. The attractiveness of high-development-level urban agglomerations extended to the entire country and not only to the province of residence. The total migrant population within the aforementioned urban agglomerations was large. Moreover, core cities, large cities, and medium cities in these agglomerations exhibited a trend of migrant population growth. Interprovincial migration predominated. Although the growth of the migrant population was large, due to the limited bearing capacity, the attraction of urban agglomerations with high level of development to the migrant population reached saturation (Fig 5D).

### Modelling determinants of migrant population distribution in China

Because of the lack of statistical data, the data of 285 prefecture-level and above cities were modelled using econometric models. The variance inflation factors (VIFs) of all independent variables were less than 10 and tolerance values were greater than 0.1 (Table 5), which indicates that independent variables were not collinear. An economic force model, a government force model, and an integrated model were applied for cities within and outside urban agglomerations, respectively. Cities within urban agglomerations were then categorised by the development level. Estimation results of econometric models are presented in Table 5.

**Table 5:** Estimation results of econometric models.

		Pooled			Outside urban agglomeration			Within urban agglomeration			Development level of urban agglomerations			
	Correlation coefficient	Coefficient	VIF	Tolerance	Economy	Government	Integrated	Economy	Government	Integrated	Low level	Lower-middle level	Upper-middle level	High level
EMP	0.627***	0.199***	2.145	0.466	0.091**	/	0.557***	0.665***	/	0.619***	0.505	0.683***	0.843***	0.663***
WAGE	0.664***	1.214***	1.940	0.515	1.247***	/	0.951***	1.894***	/	1.423***	2.073*	1.282***	1.524***	1.468**
FC	0.672***	0.075***	1.858	0.538	0.057	/	0.053	0.101***	/	0.057*	-0.165*	0.119***	0.132***	-0.020
PIV	0.759***	0.260***	3.058	0.327	0.435***	/	0.078	0.237***	/	0.222***	0.129	0.231**	0.117	0.164
EDU	0.751***	0.643***	9.951	0.100	/	0.690**	0.089	/	1.180***	0.463***	-0.652	0.275	-0.257	0.977***
SS	0.608***	-0.153*	4.609	0.217	/	-0.147	-0.136	/	-0.247**	-0.166*	0.092	-.011	-0.266*	0.031
HLTH	0.624***	-0.698***	9.207	0.109	/	-0.573*	-0.337	/	-1.191***	-0.636***	-0.433	-1.067***	0.380	-1.192***
GBX	0.816***	0.555***	9.294	0.108	/	0.673**	0.588**	/	1.278***	0.397***	1.220*	0.753***	0.004	0.351
LVL	0.635***	0.148***	2.064	0.485	/	-0.245	5.801**	/	0.248***	0.049	0.768*	0.104	0.091	-0.067
α0	/	-12.642***	/	/	-7.950**	2.894	-15.586***	-19.691***	-3.352***	-15.458***	-20.593**	-15.125***	-15.200***	-16.318***
Adjusted R^2^	/	0.834	/	/	0.459	0.322	0.582	0.847	0.785	0.862	0.935	0.840	0.835	0.890
Significance	≤0.001	≤0.001	/	/	≤0.001	≤0.001	≤0.001	≤0.001	≤0.001	≤0.001	≤0.001	≤0.001	≤0.001	≤0.001

Note:

* Significance level at 10%.

** Significance level at 5%.

*** Significance level at 1%.

All variables exhibited a significant correlation in the integrated model (Table 5), which indicates that economic and government forces jointly attracted migrant population. The adjusted *R*^*2*^ values of the economic models were higher than those of government models for cities within and outside urban agglomerations (0.847 > 0.785 and 0.459 > 0.322, respectively), suggesting that the main pull force of migration was the economy rather than the government. Moreover, integrated models’ adjusted *R*^*2*^ values were larger than government and economic models’ but smaller than the sum of adjusted *R*^*2*^ values of government and economic models for cities within and outside urban agglomerations (Table 5). This result indicates that economic and government forces interactively influenced migrants’ destination decisions.

#### Within and outside urban agglomerations

The pulling effect of economic force within urban agglomerations is greater than that outside urban agglomerations. The coefficients of EMP, WAGE, FC and PIV in the integrated model for cities within urban agglomerations were higher than those for cities outside urban agglomerations (0.619 > 0.557, 1.423 > 0.951, 0.057 > 0.053 and 0.222 > 0.078, respectively) (Table 5). Compared with cities outside urban agglomerations, migrants were more concerned with the employment scale (EMP, FC and PIV) and average wage of employees (WAGE) in the target city when making choices among cities within urban agglomerations. This result indicates the high value placed by migrants on employment opportunities and the income level in the city. According to the sixth national population census of 2010, 74.7% of interprovincial migrants aimed to obtain a better job [6]. Furthermore, in 2010, interprovincial migrants accounted for 52.35% of the migrant population in urban agglomerations and 32.72% of the migrant population outside urban agglomerations (Table 3). The major concern for migrants in urban agglomerations is to acquire well-paying jobs. Urban agglomerations, especially the core cities of urban agglomerations, have developed industries and provide numerous jobs and high wages, which represent their core attraction to the migrant population.

The pulling effect of government forces is greater outside urban agglomerations than within urban agglomerations. The coefficients of GBX and LVL in the integrated model for cities outside urban agglomerations were higher than those for cities within urban agglomerations (0.588 > 0.397 and 5.801 > 0.049, respectively) (Table 5), which reflects the quality of location-specific public service and macroeconomic regulation power of local authorities, respectively. Cities outside urban agglomerations were mainly in the developing inland western China and far away from the developed area of eastern China (Fig 2). The intraprovincial migrant population was dominant outside urban agglomerations (Table 3). This migrant population abandon the higher income and additional job opportunities because of the migration distance and cultural differences. They pay more attention to the quality of urban living than interprovincial migrants did [6]. Moreover, the administrative hierarchy (LVL) still influences the allocation of economic resources and population redistribution. Since the reform of China’s revenue-sharing system in 1994, the proportion of fiscal revenue retained by local governments has declined and the funds of fiscal transfer payment have been reallocated in a top-down manner according to the administrative hierarchy. Cities with a higher administrative hierarchy can obtain higher financial support [19]. Due to the relatively low development level of the market economy, this top-down political investment accounts for a large share of the finances of cities in developing area. The higher the administrative hierarchy, the higher is the financial autonomy and inclination of policy implementation of cities [26,42]. Therefore, the administrative hierarchy has a greater impact on the development of cities outside urban agglomerations. It is noteworthy that EDU, HLTH, and SS alone were not significantly or negatively correlated with migrant population distribution, while GBX had significantly positive relationship with migrant population distribution (Table 5), which indicates that only by improving the comprehensive service level can the city attract migrant population.

#### Urban agglomerations with different development levels

The pulling effect of economic forces increased with an upgrade in the urban agglomeration development level. Overall, the coefficients of EMP, WAGE and FC increased or became significant as the development level of urban agglomerations increased (Table 5). The proportion of interprovincial migrants among the total migrant population increased with the development level of urban agglomerations (Table 3), and the primary aims of interprovincial migrants were to obtain a better job and an increased income [6]. This result largely explains why high-development-level urban agglomerations continue to attract numerous migrants.

It is noteworthy that FC was not significant in the high-development-level model (Table 5). Because foreign investment flooded into China due to the initial low labour cost [19], the labour cost in urban agglomerations with high and upper-middle development level is higher. Therefore, foreign companies in labour-intensive industries move to cities in urban agglomerations with lower development level and directly created numerous jobs, which attracts migrants. Knowledge-intensive foreign companies stay in high-development-level urban agglomerations because they rely on the quality rather than the quantity of labour. However, knowledge-intensive foreign companies directly create less jobs than labour-intensive companies do. Another noteworthy result is that PIV was only significant in the lower-middle-development-level model. Because private enterprises and self-employed individuals are small-scale organisations. Large companies, such as listed companies and foreign transnational companies, dominated, especially in core cities such as Beijing, Shanghai, Shenzhen, Guangzhou, and Hangzhou [43]. Therefore, the proportion of jobs created by private enterprises in the high-development-level urban agglomerations is limited. Moreover, the level of market economy in low-development-level urban agglomerations is relatively low. Large state-owned enterprises dominate. Private enterprises and self-employed individuals are underdeveloped and provide limited job opportunities.

The pulling effect of government forces increases with a decrease in the urban agglomeration development level. Overall, coefficients of GBX and LVL increased or became significant as the development level of urban agglomerations decreased (Table 5). Because the proportion of intraprovincial migrant population increased with an decrease in the urban agglomeration development level (Table 3), and they place more weight on the overall location-specific amenities and social security, namely the quality of urban life in the target city [6]. Therefore, GBX became more important with a decrease in the development level. Furthermore, the absolute gap of income and job opportunities decreased with a downgrade in the development level of urban agglomerations, which weakened economic driving forces. With a decrease in the urban agglomeration development level, the development level of marketization is getting lower and market play a less and less important role in allocating the resources. On the contrary, the administrative hierarchy (LVL) has a more and more important influence on the allocation of economic resources and population redistribution.

## Conclusions and policy implications

Urban agglomerations are spatial carriers of a high concentration of essential productive factors. As one of the most active essential productive factors, migrant population gather in urban agglomerations. In this research, the spatiotemporal characteristics of migrant population distribution and the driving forces of migration in China during 2000–2010 were analysed systematically from the perspective of urban agglomerations. The results of this study are as follows: (1) Urban agglomerations are the accumulation areas of the migrant population in China, and 89.73% of the Chinese migrant population lived in urban agglomerations in 2010. (2) The attraction of urban agglomerations enlarges regional differences in migration, and people continues to migrate into urban agglomerations. (3) The interprovincial migrant population is dominant within urban agglomerations, whereas the intraprovincial migrant population is dominant outside urban agglomerations. The dominant migration mode in China will be intra-provincial migration. According to the aforementioned statistical analyses, a conceptual model of China’s migrant population distribution was developed. The evolution of the distribution pattern of the migrant population in urban agglomerations agrees with theories of regional unbalanced development, and exhibits a centralisation–decentralisation pattern.

The driving forces of migration, which shaped the spatial patterns of migrant population distribution, within and outside urban agglomerations and in different urban agglomerations were examined. Economic and government driving forces had a combined effect on migration, and the effect of economic forces exceeded that of government forces. Economic forces were more influential on migration within urban agglomerations than on migration outside urban agglomerations, which indicates that wages and job opportunities are the core attraction of urban agglomerations to the migrant population. Government forces had a higher influence on migration outside urban agglomerations than migration within urban agglomerations. Moreover, in urban agglomerations, as the development level increased, the effect of economic forces on migration increased, whereas the effect of government forces on migration decreased, because the level of market-oriented economy decreased. It should be noted that only by improving the comprehensive service level can the city attract migrant population.

Although urban agglomeration is a concept with Chinese characteristics currently, the urban agglomeration evolves from a metropolitan area [2] and has been the main spatial carrier and the core growth pole of China’s regional economic development. The development of China’s urban agglomerations can provide an example for other developing countries. Increased income and additional job opportunities are still the main pulling forces of migration. Therefore, local governments should adopt suitable investment policies to attract enterprises and provide increased job opportunities. Moreover, intraprovincial migration, who values urban life quality more, will be the dominant migration mode in China in the future. Therefore, local governments, especially those outside urban agglomerations and in less developed urban agglomerations, must improve the comprehensive public services, such as high-quality medical facilities, educational facilities and social security, to attract intraprovincial migrant workers. Moreover, Hukou system still hinders migrant population from fully access to local public service [14]. Therefore, granting Hukou or expanding the beneficiaries of public services can help them really settle down in host cities. Cities in high-development-level urban agglomerations, especially core cities, are reaching their limits of population capacity. These cities must stick to the bottom line of ecological conservation, and improve the capacity of public services and the efficiency of water usage to improve population capacity and maintain reasonable population growth. Moreover, these cities require additional highly educated skilled migrant workers to achieve industrial upgrading; therefore, they should develop targeted policies to attract skilled migrants.

China plans to launch the seventh national population census on November 1, 2020, and publish the census data at the end of 2021. Moreover, county-level cities and counties have political autonomy and economic autonomy to a certain extent. Researches at county level may unveil new characteristics. We will use these data in future research to detect latest characteristics of migrant population distribution.

## Supporting information

S1 File (PDF)
