# Supplementary material for: Spatiotemporal characteristics and determinants of internal migrant population distribution in China from the perspective of urban agglomerations
Source: PLoS One. 2021 Feb 12;16(2):e0246960. doi: 10.1371/journal.pone.0246960 (PMC7880441; doi:10.1371/journal.pone.0246960)
Supplement: S1 File — (PDF) [file pone.0246960.s001.pdf]

Request for Permission to Publish Content under CC-BY License

Dear Rights Holder or Representative,

I have submitted a paper for publication in a PLOS journal, and wish to include the content listed below in the paper. I'm hereby requesting your (or your company's or institution's) permission to include the content in my paper. Please note that all PLOS journals are published under a Creative Commons Attribution License (CC BY), which allows for unrestricted use and distribution, even commercial, as long as attribution is given to the creator or rights holder of the content. See https://creativecommons.org/licenses/by/4.0/.

To grant me permission to use the content in my PLOS paper, please fill in the information below and then scan the completed form and send it to me at my email address.

Thank you.

My name:

Ming Li

My email address:

liming57@mail2.sysu.edu.cn

Description of the content which I'm seeking permission to use (citation and/or title, and pasted screen shot, if applicable):

I map the migrant population distribution in China in 2000 and 2010 (Figure 2 and Figure 3) based on the vector data of administrative boundaries of prefecture-level city in "Spatiotemporal characteristics and determinants of internal migrant population distribution in China from the perspective of urban agglomerations".

Link to the Content:

http://www.resdc.cn/data.aspx?DATAID=201

* * *

On behalf of myself or the rights holder, I hereby grant the permission sought herein.

Signature of Party Granting Permission:

Qiang Wang , RESDC

Date:

2020 Dec 7

Printed Name and Title:

We grant you permission to use the administrative boundaries of prefecture-level city in China in 2015 in your PLOS paper.
